# Modeling of high voltage induction motor cooling system using linear regression mathematical models

**DOI:** 10.1371/journal.pone.0276142

**Published:** 2022-11-29

**Authors:** Nurfatihah Syalwiah Rosli, Rosdiazli Ibrahim, Idris Ismail, Madiah Omar

**Affiliations:** 1 Department of Electrical & Electronics Engineering, Universiti Teknologi PETRONAS, Seri Iskandar, Perak, Malaysia; 2 Department of Chemical Engineering, Universiti Teknologi PETRONAS, Seri Iskandar, Perak, Malaysia; Southwest Jiaotong University, CHINA

## Abstract

Achieving reliable power efficiency from a high voltage induction motor (HVIM) is a great challenge, as the rigorous control strategy is susceptible to unexpected failure. External cooling is commonly used in an HVIM cooling system, and it is a vital part of the motor that is responsible for keeping the motor at the proper operating temperature. A malfunctioning cooling system component can cause motor overheating, which can destroy the motor and cause the entire plant to shut down. As a result, creating a dynamic model of the motor cooling system for quality performance, failure diagnosis, and prediction is critical. However, the external motor cooling system design in HVIM is limited and separately done in the past. With this issue in mind, this paper proposes a combined modeling approach to the HVIM cooling system which consists of integrating the electrical, thermal, and cooler model using the mathematical model for thermal performance improvement. Firstly, the development of an electrical model using an established mathematical model. Subsequently, the development of a thermal model using combined mathematical and linear regression models to produce motor temperature. Then, a modified cooler model is developed to provide cold air temperature for cooling monitoring. All validated models are integrated into a single model called the HVIM cooling system as the actual setup of the HVIM. Ultimately, the core of this modeling approach is integrating all models to accurately represent the actual signals of the motor cooler temperature. Then, the actual signals are used to validate the whole structure of the model using Mean Absolute Percentage Error (MAPE) and Root Mean Square Error (RMSE) analysis. The results demonstrate the high accuracy of the HVIM cooling system representation with less than 1% error tolerance based on the industrial plant experts. Thus, it will be helpful for future utilization in quality maintenance, fault identification and prediction study.

## I. Introduction

A high voltage induction motor (HVIM) is widely used for operations of heavy industry due to its high reliability and efficiency. HVIM is a three-phase induction motor that is usually used in heavy industry due to its robustness, efficiency, reliability and less expensive. Thus, the HVIM motor is designed to operate with high voltage ranging from 6kV and above. High power of motor usually uses induction motor type [1]. Generally, a high voltage induction motor is provided with an external cooling system to maintain the motor efficiency. However, HVIM also experiences mechanical, electrical, and external faults during the running time and exposes it to degradation that increases with time. Furthermore, it experiences a heavy-duty cycle and overheating. The failure of HVIM will result in entire system downtime if they are located in a vital position in the plant. Undetectable faults may lead to sudden failure which causes significant economic losses. Without proper maintenance of the motor cooler, it will affect the performance of HVIM. Hence, the motor can be overheating due to the increase in temperature and accelerate the degradation of the motor [2]. Finally, the higher temperature of the motor will stop the operation and cause a sudden shutdown and it may cause a major economic loss. Therefore, a model that reflects the actual behavior of the HVIM cooling system is needed to represent the dynamic model of the system.

Conventionally, the induction motor electrical and thermal analysis is developed separately to represent the thermal behavior of HVIM [3]. The electrical equivalent circuit of the motor is developed first to provide losses. Then, the thermal model is obtained from the thermal losses of the heat generation for temperature parameter calculation. The steady-state of an induction motor thermal model is directly dependent on the design characteristics, geometric dimensions, and cooling technology used. Furthermore, the accuracy of the mathematical model of thermal model calculations for different motors will yield varied results, both quantitatively and qualitatively. As a result, relying on the individual model for the thermal analysis in an electrical machine will be inaccurate and unreliable [4]. Thus, it is important to construct the combined model and analyze it to prove the conceptual design. Integration of all models at the preliminary design level will helps to predict the thermal condition of an induction motor during operation with better accuracy. Checking the adequacy of the mathematical model should be carried out according to the actual data obtained from the actual plant. Therefore, the development of the electrical, thermal and cooler model integration approach is proposed to represent the actual behavior of motor cooling system.

Most of the works focused on developing an electrical model of induction motor using the Direct Quadrate (DQ) model. For example, DQ mathematical model development for electrical fault analysis of squirrel cage induction motor was done by Pema [5] and Rahaman [6]. They applied the DQ model to describe the behavior of the three-phase induction motor. Mathematical modeling using the DQ model is widely implemented due to its capability to replicate any induction motor operation from the simplified equation with structural symmetry [1, 7, 8]. A review has been conducted in [7] to describe the advantages and disadvantages of mathematical models for induction motors studies.

Many works are reported to investigate the thermal analysis of a three-phase induction motor using dynamic simulation based on a mathematical model. There are three categories reported in developing an induction motor model and its cooling system which are model-based [9], data-driven [10] and hybrid [11] methods. Currently, there are some works reported in utilizing the data-driven model in induction motor modeling. A data-driven model is applicable when there is an availability of a large amount of historical data. Most of the studies focus on fault diagnosis of induction motors. For instance, Razvan [12] applied the Nonlinear Autoregressive Network with Exogenous (NARX) method whereas Hai Guo [13] proposed a deep neural network for temperature prediction of the motor. However, deficiency in historical faulty data became the drawback of developing an efficient data-driven model. Model-based is usually uses mathematical models that are derived from thermodynamics and physical laws of the system. For example, the previous works have been done by Naskar [14] and Karashima [15] using the Finite Element Method (FEM) to monitor the temperature of the stator and rotor. Although FEM has high estimation accuracy, it has some drawbacks compared to Lumped Parameter Thermal Model ((LPTM) which relies on the information of material properties, motor dimension and boundary conditions, leading to complex calculation and high computational times [16]. However, major studies have emphasized motor power of less than 1 Mega Watt that uses the air-to-air cooling method which is not suitable for heavy industrial applications. Moreover, the previous research is based on the simulated fault, and the design stage of the cooler does not reflect the actual condition in industry application. Besides that, the model development using FEM is difficult to implement because it involves many parameters to calculate the temperatures of induction motor elements [17]. Besides, most studies have focused on the design improvement of the air-to-air type of cooling system for low and medium-power motors where it took a long time to implement and solve the thermal FEM models of electric motors.

Conventionally, a mathematical model of the heat exchangers (cooler) can be constructed using methods like the effectiveness-number of transfer units (e-NTU), logarithmic mean temperature difference (LMTD), and others. Belusko and Bruno looked into the effectiveness-NTU approach for flat plate phase change unit design and Tay et al. created a mathematical model for radial-fin-tube and tube-in-tank tank systems [18]. However, these models have limits since they rely on heat exchanger characteristics. Another mathematical model that can be derived from the thermodynamics equation to model the heat exchanger of the motor cooler is using LMTD, heat transfer theory and energy balance theory [19, 20]. This method is simpler, more convenient and feasible to develop an HVIM cooler model with current data availability. The solution to the shortcut and a simple model is by modifying the LMTD equation with a correction factor adopted from the concept of e-NTU to match the detailed heat exchanger model. The details of the motor cooler model development will be discussed in the next section. However, some assumptions need to be identified to develop the physical model of the motor cooler.

**Fig 1:**
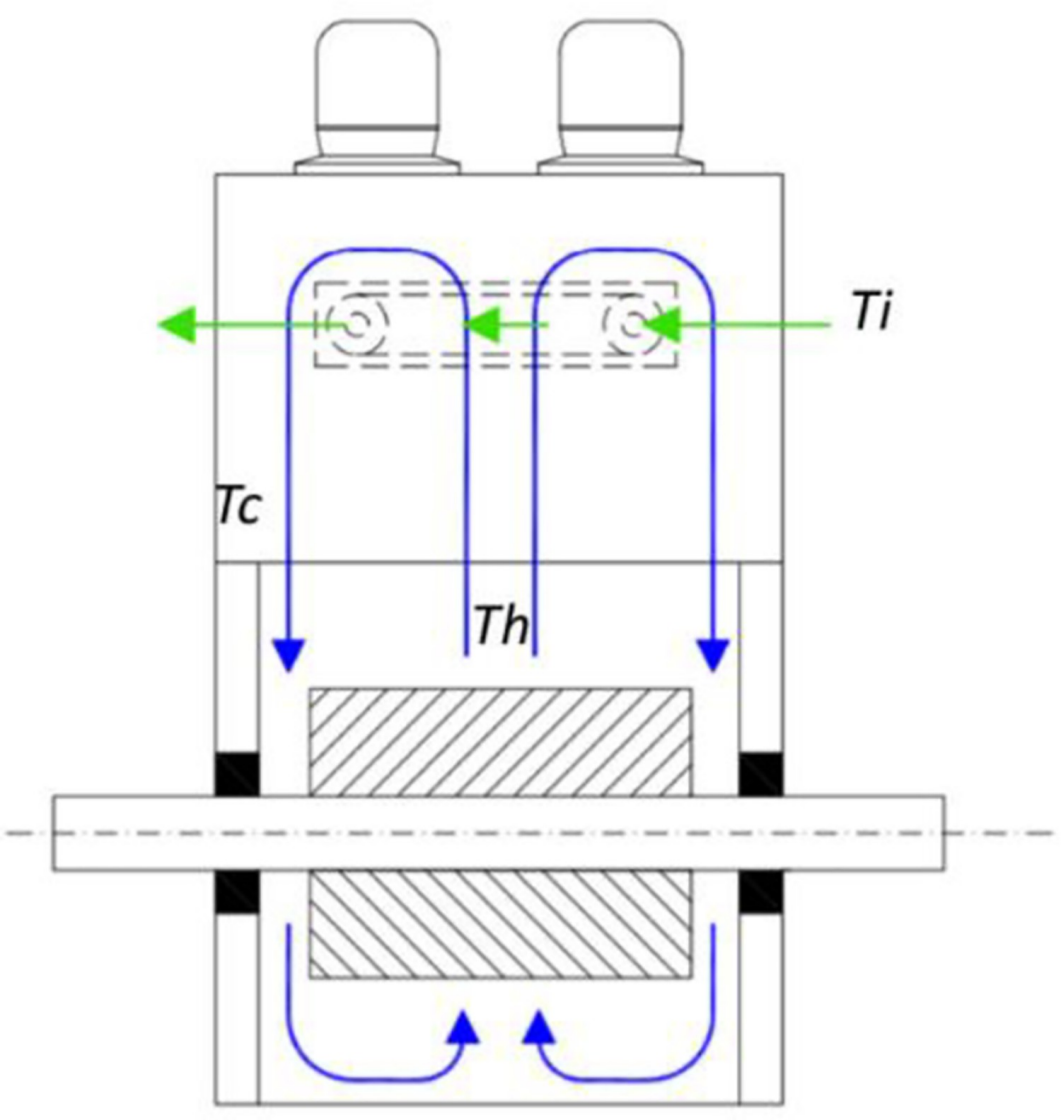
Motor cooler architecture.

Therefore, a model that reflects the actual behavior of the HVIM motor cooler is necessary. Hence, the combined mathematical and linear regression models are utilized in this study to represent the HVIM cooling system for better analysis of thermal management. Besides that, this model can give useful information for prognosis output by merging mechanistic knowledge of the system, equation of defect growth and condition monitoring data. The architecture of the motor cooler is illustrated in Fig 1.

A cooler of HVIM is a significant component that transfers heat from water to air passing through the cooler finned tube as shown in Fig 1. Commonly, the HVIM cooling system is using Totally Enclosed Water to Air Cooled (TEWAC) [21], and the plate-fin cooler is usually used [18, 22]. The cooler’s fins are normally made of aluminum, and the cooled water is circulated through a tube. Water is forced through the fins soldered to the aluminum tubes to achieve heat exchange. The cooler must continue to ensure efficient heat transfer and produce heat exchange from the cooler with the motor, regardless of operating conditions or ambient temperature [23, 24].

The water-to-air cooling system removes heat from the hot air, *T*_*h*_ to produce cold air, *T*_*c*_. When there is a temperature difference between two or more mediums, heat transfer happens. Conduction, convection, and radiation are the three basic modes of heat transfer. The mechanisms of conduction and convection are in charge of dissipating the heat generated during combustion throughout the motor. The radiation is neglected in this study due to the very small effect on this cooling system. Correspondingly, heat is delivered to the cooling coolant by conduction and convection. At last, these two processes describe how ambient air cools the system and how it interacts with the cooler to disperse heat created in the system to the surrounding air [25].

The work of this paper presents the simulation of the electrical, thermal and cooler models of a high voltage induction motor. Actual data from one of the industrial companies in Malaysia that use HVIM to drive the compressor are taken to validate the mathematical models. This data was gathered with daily time-series data. The proposed of these models’ integration including the cooling system is the main technical contribution of this work.

## II. Mathematical model of HVIM

### A. Electrical model

**Fig 2:**
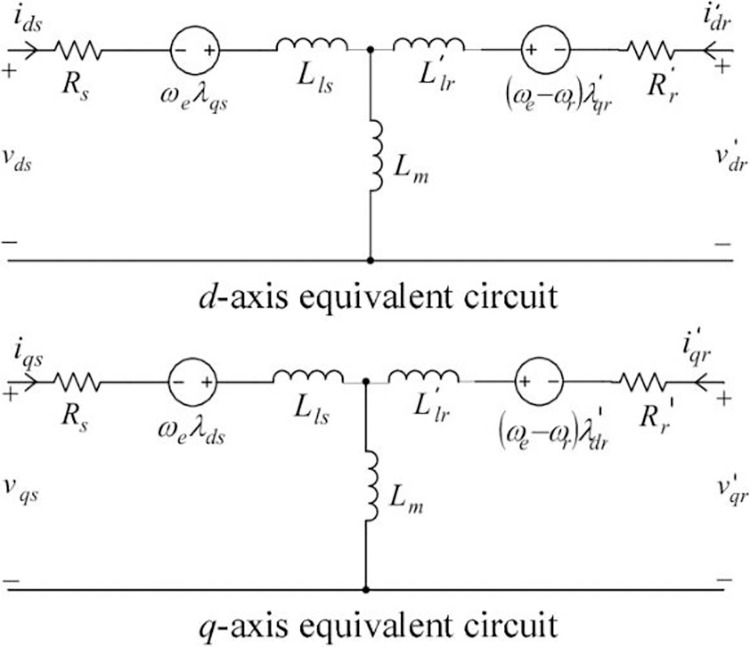
Equivalent circuit of DQ model.

The electrical model of HVIM is developed based on the equivalent circuit of the DQ model as shown in Fig 2.

In the DQ model, park transformation has been applied by transforming the voltage and torque equations from the ABC frame to the DQ frame [6]. DQ model provides a convenient way of modeling the motor and it is the most appropriate for numerical solutions. Torque has given an impact on voltage and slip inputs in an electrical model. However, in this model, the torque is assumed constant at the steady-state. Therefore, the mechanical model is not required in this study.

The DQ model converts the three-phase voltage into two axes, The stator voltage equations are defined by

$$v_{qs}=R_{s}i_{qs}+\frac{{d\lambda }_{qs}}{dt}+\omega_{e}{d\lambda }_{ds}$$ (1)

$$v_{ds}=R_{s}i_{qr}+\frac{{d\lambda }_{ds}}{dt}-\omega_{e}{d\lambda }_{qs}$$ (2)

$$v_{0s}=R_{s}i_{0s}+\frac{{d\lambda }_{0s}}{dt}$$ (3)

where *v*_*qs*_, *v*_*ds*_, and *v*_0*s*_ are the d-axis, q-axis, and zero-sequence stator voltages.

The rotor voltages are defined by

$$v_{qr}^{\prime }=R_{r}^{\prime }i_{qr}^{\prime }+\frac{{d\lambda }_{qr}^{i}}{dt}+(\omega_{e}-\omega_{r})\lambda_{dr}^{\prime }$$ (4)

$$v_{dr}^{\prime }=R_{r}^{\prime }i_{dr}^{\prime }+\frac{{d\lambda }_{dr}^{i}}{dt}-(\omega_{e}-\omega_{r})\lambda_{qr}^{\prime }$$ (5)

Electromagnetic torque is defined as in (6).


$$T_{e}=1.5*p*(\lambda_{ds}i_{qs}+\lambda_{qs}i_{ds})$$ (6)

The equations of magnetic flux of stator and rotor are:

$$\lambda_{qs}=L_{s}i_{qs}+{L_{m}i}_{qr}^{\prime }$$ (7)

$$\lambda_{ds}=L_{s}i_{ds}+{L_{m}i}_{dr}^{\prime }$$ (8)

$$\lambda_{qr}^{i}=L_{r}^{\prime }i_{qr}^{i}+L_{m}i_{qs}$$ (9)

$$\lambda_{dr}^{i}=L_{r}^{\prime }i_{dr}^{i}+L_{m}i_{ds}$$ (10)

The inductance of stator and rotor are described as the following equation.


$$L_{s}=L_{ls}+L_{m}$$ (11)

$$L_{r}^{\prime }=L_{lr}^{\prime }+L_{m}$$ (12)

A few assumptions were made in constructing the electrical model of induction motor which are (i) the model does not include a representation of the saturation of leakage fluxes, (ii) rotor bars are short circuit and there is no load and (iii) the motor is symmetrical and sinusoidal distributed. The parameters used in designing the induction motor are given in the following Table 1.

**Table 1:** Parameter setting for HVIM.

Parameter	Symbol	Unit	Value
Nominal power	*S*	MW	7
Nominal input voltage (line voltage)	*V*	kV	11
Stator nominal current	*I*_*s*_	A	433
Nominal stator frequency	*f*_*s*_	Hz	50
Synchronous Speed	*ω*_*s*_	rpm	1478
Number of poles	*P*	-	4
Nominal power factor	*pf*	-	0.8
Inertia	*J*	kgm^2^	270
Stator Resistance	*R*_*s*_	Ω	0.1037
Rotor Resistance	*R*_*r*_	Ω	0.2593
Stator Inductance	*L*_*s*_	H	0.0077
Rotor Inductance	*L*_*r*_	H	0.00396
Mutual Inductance	*L*_*m*_	H	0.1903
Torque	τ	Nm	44520

These parameters are fed into a MATLAB simulation of an induction motor to obtain the output current that will be used in the development of a thermal model. The output of the electrical model which is the stator current will be used as input to the thermal model to compute the temperature of the motor.

### B. Thermal model

A thermal model is required to represent the motor temperature. The motor temperature can be gained from the copper losses equation, as it is produced from the current flow in the stator and rotor conductors. The losses can be computed from the equation *I*^2^*R*, where *R* is the circuit resistance which varies with temperature and frequency [26]. While motor temperature can be computed from the stator copper loss by measuring the stator current and resistance of the winding at the desired temperature. The equation is developed based on the derivation of power loss with the heat dissipation equation [27]. For developing a thermal model, the input current and resistance are required to calculate the motor temperature. The current is provided from the electrical model of the induction motor, whereas resistance is obtained from (13).

$$R=R_{s}(1+\alpha (T_{w}-T_{a}))$$ (13)

where,

*R*_*s*_ = stator resistance at ambient temperature

*a* = temperature coefficient of resistance

*T*_*a*_ = ambient temperature

*T*_*w*_ = output motor temperature (winding temperature)

The temperature coefficient of resistance, *a* represents the resistance change factor per degree of temperature change in a material. It should be noted that the assumption was made that the heat loss is negligible. Therefore, the differential equation for motor temperature can be represented as (14).

$$I^{2}R=mc_{p}\frac{dT_{w}}{dt}$$ (14)

where *m* is the mass of the motor stator and *c*_*p*_ is the specific heat capacity of motor steel. After that, (14) is integrated to obtain the motor temperature as described in (15).


$$T_{w}=\frac{1}{mc_{p}}\int_{0}^{t}I^{2}R.dt$$ (15)

The thermal model parameter consists of stator current, resistance, ambient temperature, and several constant values. The value of constants to develop the thermal model is given in Table 2.

**Table 2:** Parameter assumption for thermal model development.

Parameter	Symbol	Unit	Value
Heat transfer coefficient of steel	*c*_*p*_	J/kg-K	481
Mass of stator	*m*	kg	7300
Stator resistance	*R*_*s*_	Ω	0.1037
Ambient temperature	*T*_*a*_	°C	27
Temperature coefficient of resistance	*a*	°C^−1^	3.93× 10^−3^

Hot air temperature (*T*_*h*_) is important in the development of motor coolers. However, there is a limitation in obtaining the rate of heat dissipation when cooling occurs, since it consists of an unknown value of *Aλ*, as shown in Eq (16).


$$Q_{h}=A\lambda \theta$$ (16)

Where *Q*_*h*_ is the rate of heat dissipation from the cooling medium, *A* is the area of the motor surface, *λ* is the rate of heat dissipation from the motor surface and θ is the temperature difference between the winding temperature, *T*_*w*_ and hot air temperature, *T*_*h*_. The thermal model is solved by rearranging Eq (16) to obtain *T*_*h*_ as expressed in Eq (17).


$$T_{h}=T_{w}-\frac{Q_{h}}{A\lambda }=f(T_{w})$$ (17)

$-\frac{Q_{h}}{A\lambda }$ can also be simplified as *θ* a temperature rise coefficient. Finally, the output of the thermal model, *T*_*h*_ is obtained by substituting Eq (15) into Eq (17) as illustrated in Eq (18).


$$T_{h}=\frac{2}{mc_{p}}\int_{0}^{t}I^{2}R.dt-f(T_{w})$$ (18)

Since the rate of heat dissipation in temperature rise, *Aλ* is not available, $\frac{Q_{h}}{A\lambda }$ is approximated based on an equation *f*(*T*_*w*_) using regression methods for developing the thermal model. Thus, the correlated variable with the *T*_*h*_ is the *T*_*w*_ which is applied as the input for the model development. Data samples of 70% training data and 30% validation data are prepared. Linear regression, polynomial regression, quadratic SVM, and GPR are utilized in the modeling. Numerical and graphical methods are used to analyze the performance of the models. The prediction and residual error plots are displayed to view the randomness and unpredictability of the model. The residual error, *e*_*r*_ is calculated by subtracting the predicted value, *y*_*p*_ from the observed value, *y*, as presented in (19).


$$e_{r}=y-y_{p}$$ (19)

The models are also evaluated using R-squared which statistically quantifies how close the data is to the fitted line with a best-fit value of 1. Lastly, the high accuracy model is selected to represent the hot air temperature variable in the thermal model. Then, the whole development of the thermal model is developed in the MATLAB Simulink program. The output of the thermal model which is hot air temperature is subsequently cooled by the motor cooler. Then, this parameter is prepared for HVIM cooler model development.

### C. Cooler model

The HVIM cooling system removes heat from the hot air to produce cold air. When there is a temperature difference between two or more mediums, heat transfer happens. Conduction, convection, and radiation are the three basic modes of heat transfer. The equations are developed based on the theory of heat transfer [28]. The mechanisms of conduction and convection are in charge of dissipating the heat generated during combustion throughout the motor. The radiation is neglected in this study due to the very small effect on this cooling system. The temperature differential between the hot air input, *T*_*h*_ and cold air, *T*_*c*_ outlet terminals can be used to calculate the heat loss through the cooler tube, where the cold air temperature is considered to be the same as the water cooler temperature. ${\dot{Q}}_{total}$ is $\frac{dQ}{dt}$ where the total heat transfer from the hot air temperature to the water cooler. It is the total cooling achieved by the hot air temperature. It can be calculated as the Equation below:

$${\dot{Q}}_{total}=\dot{w}C_{p}(T_{h}-T_{c})$$ (20)

The heat energy transferred in the water cooler is using the concept of the heat exchanger. The heat transfer through the motor cooler is using the equation below;

$${\dot{Q}}_{h-i}=U\times A\times (F)LMTD$$ (21)

where LMTD is the temperature difference between the hot air and cold air at the inlet and outlet of the motor cooler. *U* is the heat transfer coefficient with the unit of W/(m^2^K) and *A* is the area of the motor cooler measured in m^2^. *F* is the correction factor for *LMTD* values modification based on the e-NTU concept. The *F* and *LMTD* equations are defined as follows in Eqs (22) and (23) respectively;

$$F=\frac{T_{c}-T_{i}}{T_{h}-T_{i}}$$ (22)

$$LMTD=\frac{\left(T_{h}-T_{i})-(T_{c}-T_{i}\right)}{ln[((T_{h}-T_{i})/(T_{c}-T_{i})}$$ (23)

where *T*_*i*_ is the water cooler temperature. The correction factor is added to adjust the shortcut cooler model by providing a linear update and matching the detailed cooler model.

The heat transfer between two surfaces that are at different temperatures is known as conduction. The conduction through the motor cooler wall is very small. Therefore the amount of heat transfer between the cooler tube walls can be neglected. Thus, ${\dot{Q}}_{i-i}$ is the rate of heat loss through the cooler wall by conduction is approximately equal to zero as shown in the equation below;

$${\dot{Q}}_{i-i}\approx 0$$ (24)

The heat transfer to the outside cooler will decrease the temperature of hot air and become cold air. The rate of heat transfer from the cooler wall to the air is determined by the following equation;

$${\dot{Q}}_{i-c}=wC_{p}\frac{dT_{c}}{dt}$$ (25)

Based on the thermodynamics law, the energy balance equation is obtained as follows:

$${\dot{Q}}_{total}={\dot{Q}}_{h-i}+{\dot{Q}}_{i-i}+{\dot{Q}}_{i-c}$$ (26)

After substituting (21), (25) and (26) into (27), the overall equation will be:

$$\dot{w}C_{p}(T_{h}-T_{c})=U\times A\times (F)LMTD+wC_{p}\frac{dT_{c}}{dt}$$ (27)

The above equation needs to rearrange to get the output of the motor cooler which is cold air temperature. The equation that we have after moving $\frac{dT_{c}}{dt}$ to the left side is

$$\frac{dT_{c}}{dt}=\left(\frac{\dot{w}C_{p}(T_{h}-T_{c})+U\times A\times (F)LMTD}{wC_{p}}\right)$$ (28)

The cold air temperature is gained by integrating the whole equation.


$$T_{c}=\int \left(\frac{\dot{w}C_{p}(T_{h}-T_{c})+U\times A\times (F)LMTD}{wC_{p}}\right)dt$$ (29)

The final equation will be used to design the motor cooler model. However, there are some assumptions in developing a motor cooler model using Simulink-MATLAB:

- The water temperature of the inlet and outlet leaving the system is the same.
- The temperature does not affect any of the constants.
- There is no energy accumulation in the cooler tube wall material.
- Water in cooler tubes is well-mixed and incompressible in both radial and axial dimensions.
- The water mass flow rate of entering and leaving is the same.
- The value of constants are given in Table 3 below:

**Table 3:** Parameter assumption for motor cooler model.

Parameter	Symbol	Unit	Value
Heat transfer coefficient	*U*	W/(m2K)	800
Motor Cooler Area	*A*	m2	300
Water cooler flowrate	*ẇ*	kg/s	7200
Specific heat capacity	*c*_*p*_	kJ/kg-K	1.8
Heat transfer coefficient	*U*	W/(m2K)	800

Tabular and graphical results from the transient to steady state simulation will be presented for a clearer comparison of the results obtained. Two performance indicators are employed to assess the model’s effectiveness in this study: mean absolute percentage error (MAPE) and root mean square error (RMSE). MAPE is computed by dividing the total number of absolute errors by the demand. It is calculated as the average percentage of errors. While RMSE is a prominent Key Performance Indicator (KPI) for measuring forecast accuracy. The equations to calculate RMSE and MAPE are described as follows:

$$RMSE=\sqrt{\frac{1}{N}\sum_{i=1}^{N}{\left(y_{p}-y\right)}^{2}}$$ (30)

$$MAPE=\frac{1}{N}\sum_{i=1}^{N}\frac{\left|y_{p}-y\right|}{y}\times 100\%$$ (31)

The model calculates the error between the expected value (*y*_*p*_) and the actual value (*y*) in each training and validation iteration for *N* set data number. The evaluations of the models’ performance are based on the lowest evaluation metrics. These evaluation metrics are used throughout the performance evaluation.

## III. Results and discussions

Due to the complexity of the system’s structure, using mathematical equations to accurately depict the real process with an acceptable error tolerance for incomplete or damaged data is extremely difficult. Therefore, to obtain the cooler parameters, the development of a motor cooler model is required. Since the input of the motor cooler is hot air temperature, the thermal model of the motor must be built. Whereas, the input of the thermal model is motor current. For that reason, an electrical model must be developed first to provide current data followed by a thermal and cooler model. The simulation results of each model are discussed in their respective subsections.

### A. Electrical model

**Fig 3:**
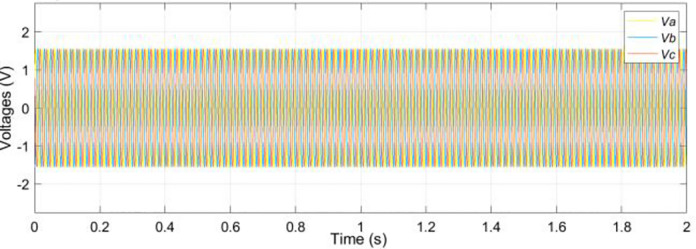
Phase voltage.

**Fig 4:**
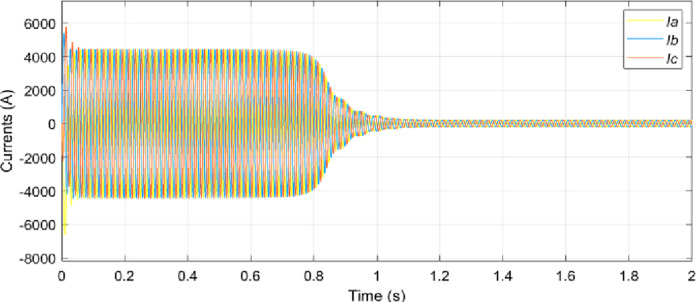
Stator phase current.

**Fig 5:**
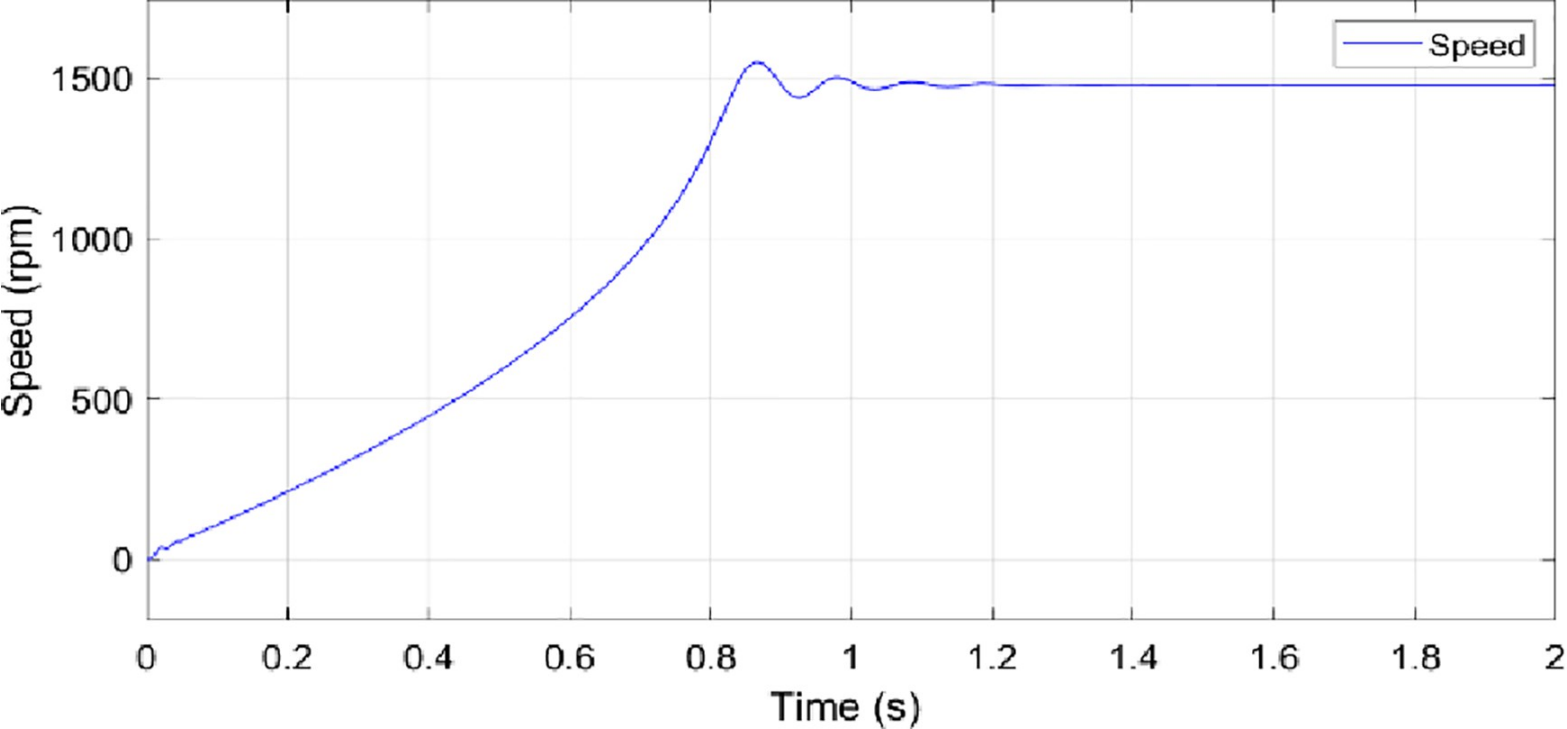
Speed.

**Fig 6:**
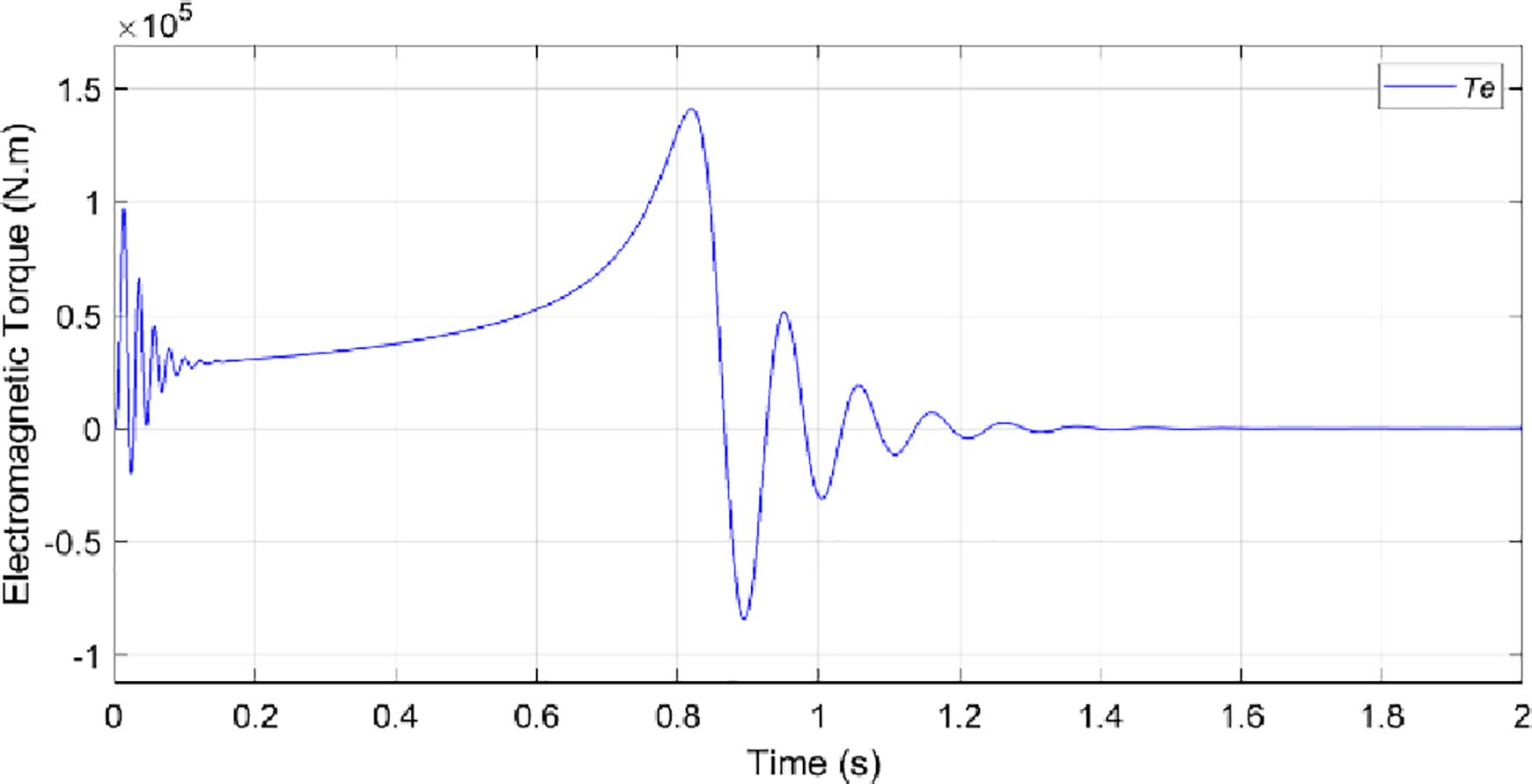
Electromagnetic torque.

The electrical sub-model in a generalized dynamic model of an induction motor is used to implement the DQ model conversion of three-axis to two-axis stator voltage to calculate current and electromagnetic torque [29]. Aside from that, the rotor speed is calculated using the torque balance equation while omitting the friction. The simulation of the model is conducted based on the calculated parameters listed in Table 1. Figs 3–6 depicts the simulation results of the phase voltages, stator phase currents, rotor speed, and electromagnetic torque respectively.

**Fig 7:**
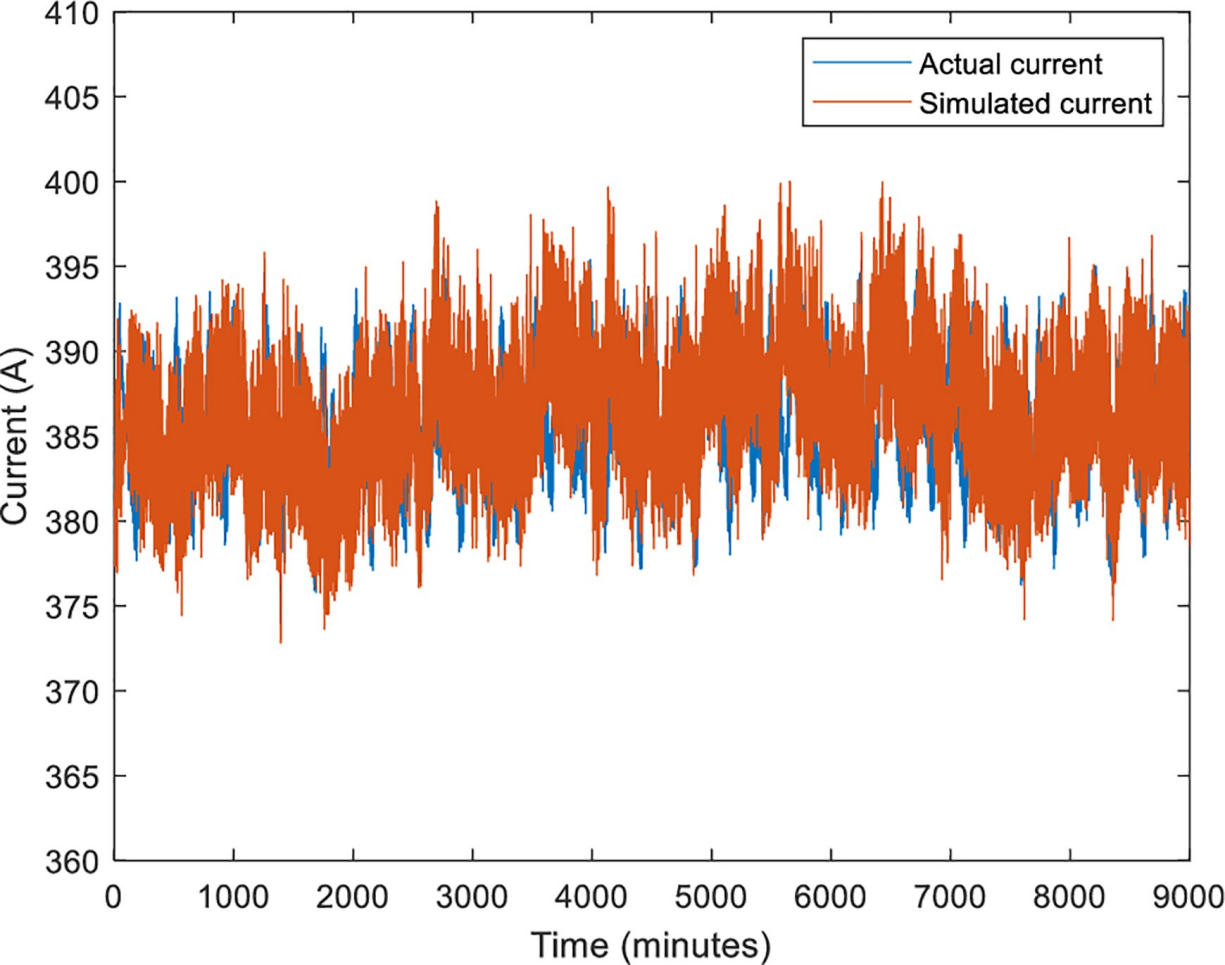
Comparison between actual and simulated current.

In the no-load condition, the motor starts from a standstill and reaches a steady maximum speed of 1478 rpm as shown in Fig 5. The value of the starting current, as well as the value of the starting torque, is highest at the positive pulse during start-up. On other hand, when the motor is running at its permanent mode, the root mean square values of the stator currents *I*_*a*_, *I*_*b*_ and *I*_*c*_ are 367.5 A as depicted in Fig 6. Besides that, it can be observed that the speed and current reach steady-state at around 1.2s. Fig 4 presents the currents that vary until they reach the synchronous speed at which they are equal to zero. The motor also exhibits pulsating torque at the beginning but the average torque is positive and helps to overcome the inertia torque. After that, it rapidly drops when it reaches synchronous speed and decreases until it reaches a steady-state of zero torque. Thus, the simulated responses from the DQ modeling of the induction motor have provided satisfactory results in terms of torque and speed characteristics. Therefore, this method is applied to provide the behavior of the HVIM. The actual voltage is fed into the electrical model to simulate the current trend. The simulated current is plotted together with the actual current to verify the performance of the three-phase induction motor model as illustrated in Fig 7.

**Fig 8:**
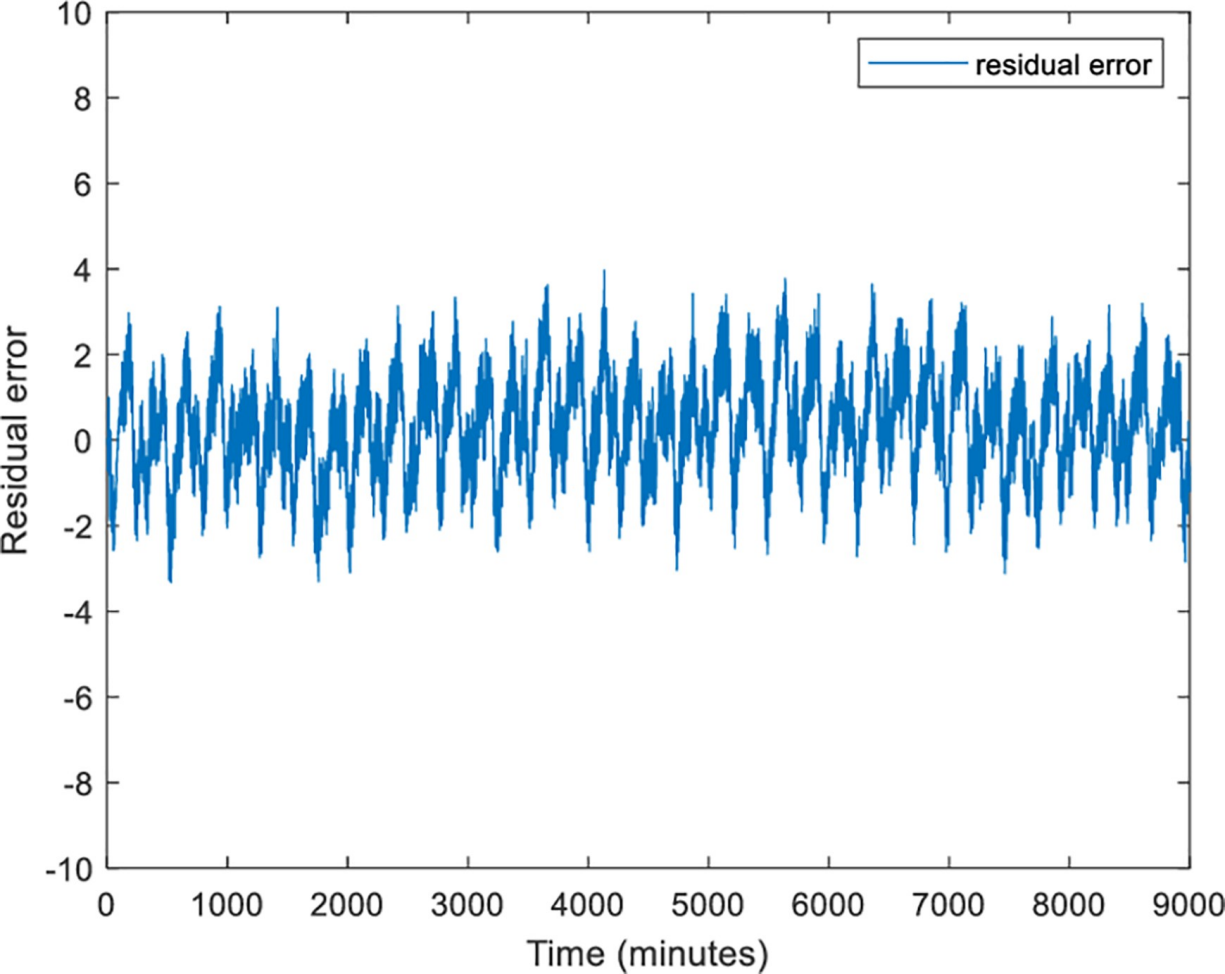
The residual error between the actual and simulated current.

The actual voltage is fed into the electrical model to simulate the current trend. The simulated RMS current is plotted together with the actual RMS current to verify the performance of the three-phase induction motor model as illustrated in Fig 7. Based on this figure, the observed response reveals the identical trend replication of actual current data. The signals are almost overlapped with each other. To evaluate the simulated response, the residual plot analysis is presented in Fig 8.

Based on Fig 8, the errors are lies in the range of [–4,4] with a nearly identical cycle. This is might be related to the uncertainty issue of the induction motor parameter [30]. The uncertainty error is regularly distributed, which indicates that the range of its contributions is the same. The uncertainty might occur in modeling due to approximation uncertainty, model uncertainty, or input uncertainty. However, the residual patterns are repetitive and not random. The percentage error and RMSE analysis for the simulated response, when compared to the actual response, are 0.872 and 4.795, respectively. The percentage error is still below 1% and the RMSE is considered low for the development of the electrical model. Therefore, it can be concluded that the simulation of the three-phase induction motor is successfully implemented by verifying it with real data. In addition, the proposed model also can describe the behavior of the system magnificently.

### B. Thermal model

The thermal model is developed and presented to indicate how temperature rise affects by the stator resistance value. The value of stator resistance increases as the motor temperature increases. Then, the winding temperature, *T*_*w*_ is obtained from the heat transfer equation and power loss equation. After that, the thermal model is integrated with the *T*_*h*_ model estimation, *f*(*T*_*w*_) using linear regression and the winding temperature data as the input.

**Fig 9:**
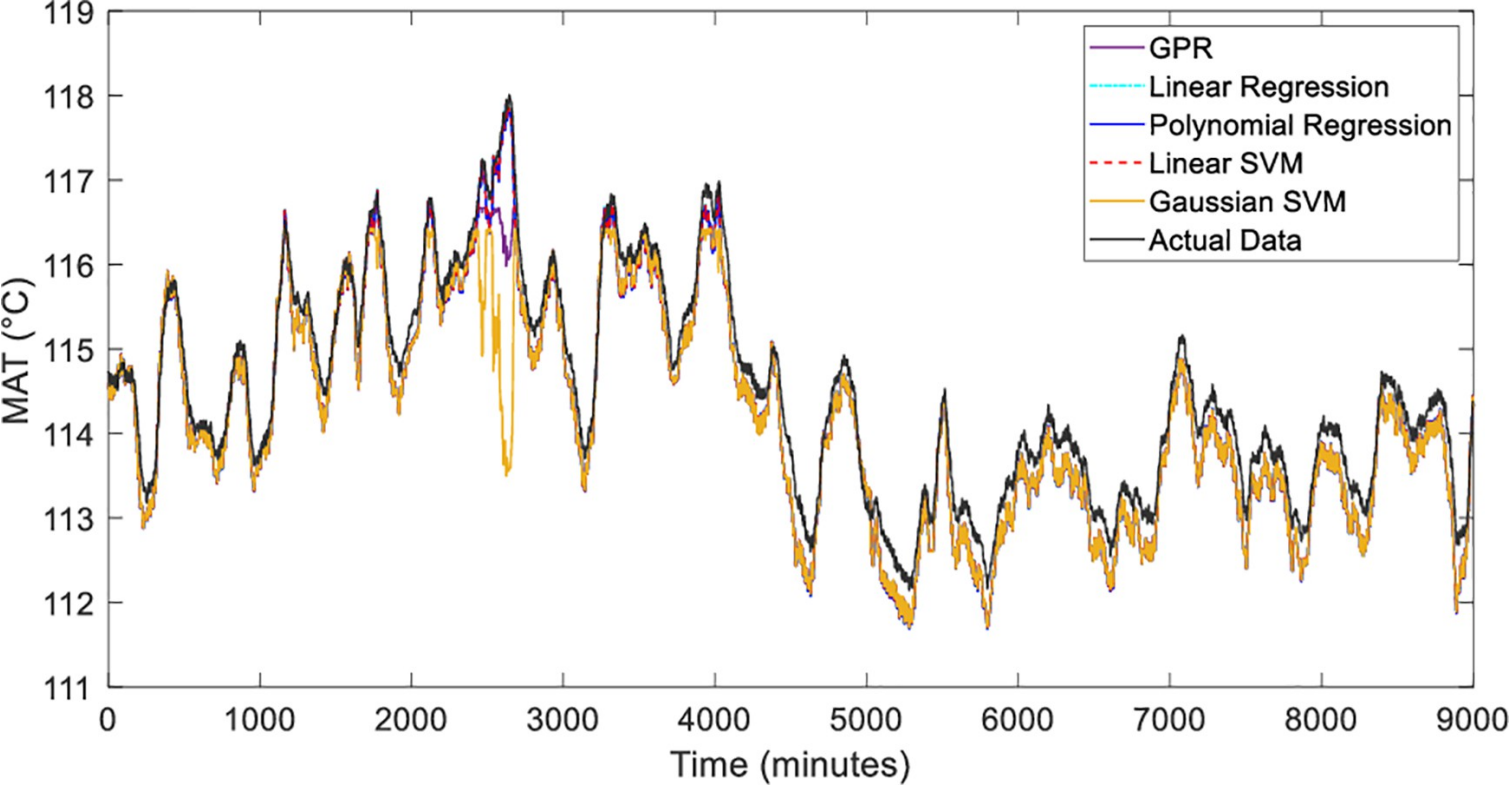
*T*_*h*_ response of five estimation methods without zooming.

**Fig 10:**
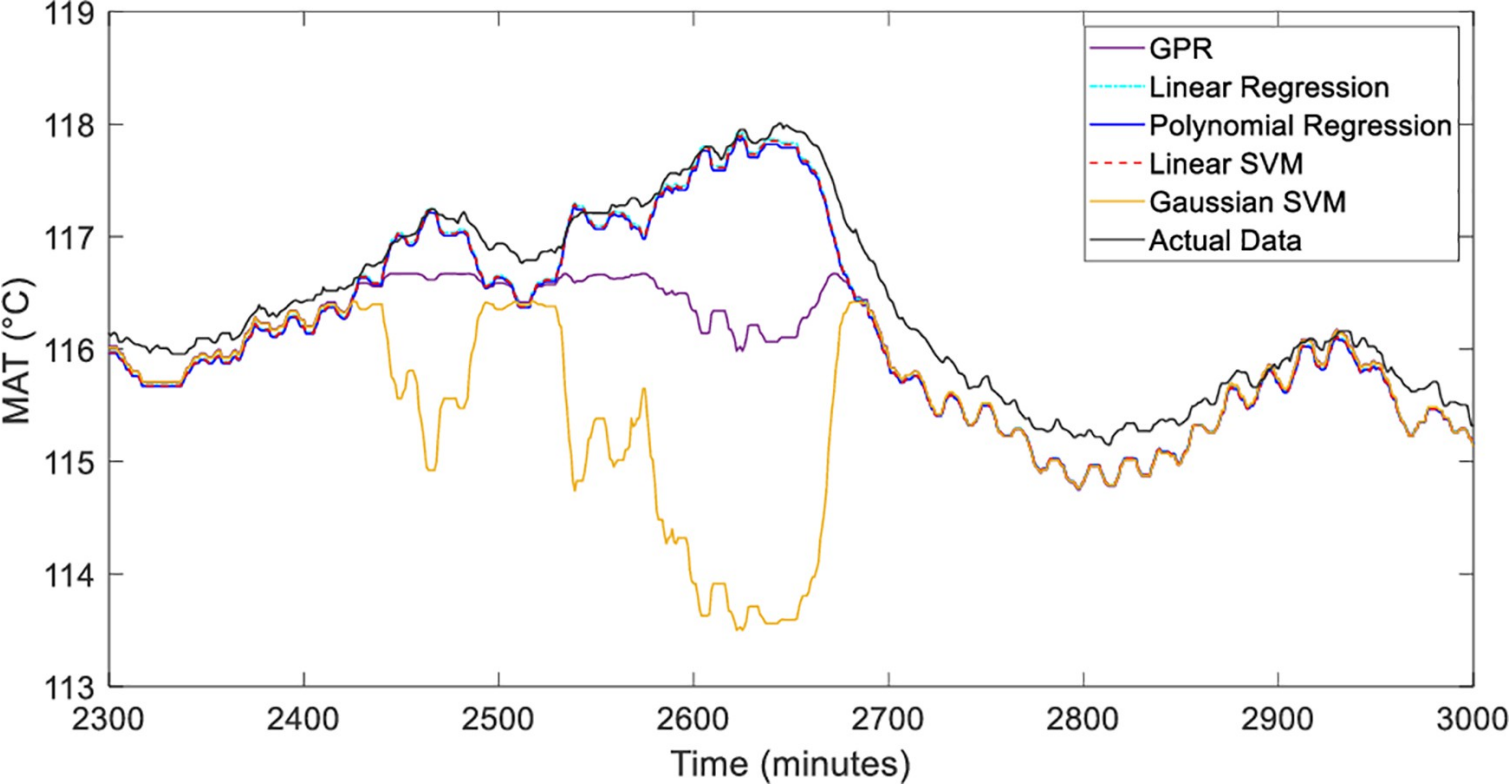
*T*_*h*_ response in zoomed-in between 2300 and 3000 minutes.

The five estimation methods of Linear Regression [31], Polynomial Regression [32], Gaussian Process Regression (GPR) [33], Linear SVM [34] and Gaussian SVM [35] are analyzed using graphically and numerically. Fig 9 shows the performance of the motor temperature model with the input of winding temperature, while Fig 10 depicts the magnified graph during the dynamic temperature changes during operations. The black line represents the actual data from the plant. With careful observations, the methods were able to capture the actual trend. However, Gaussian SVM and GPR techniques are slightly deviated during the high peak of the day starting from 2300 minutes to 2700 minutes. The Linear Regression model is identified as the best representation for the model followed by Linear SVM. This is due to the strong linear relationship between hot air temperature and winding temperature. The capability of Linear SVM also to maximize the margin and minimize the regression error using the linear kernel parameter around the pre-determined hyperplane can provide a good prediction [36].

**Fig 11:**
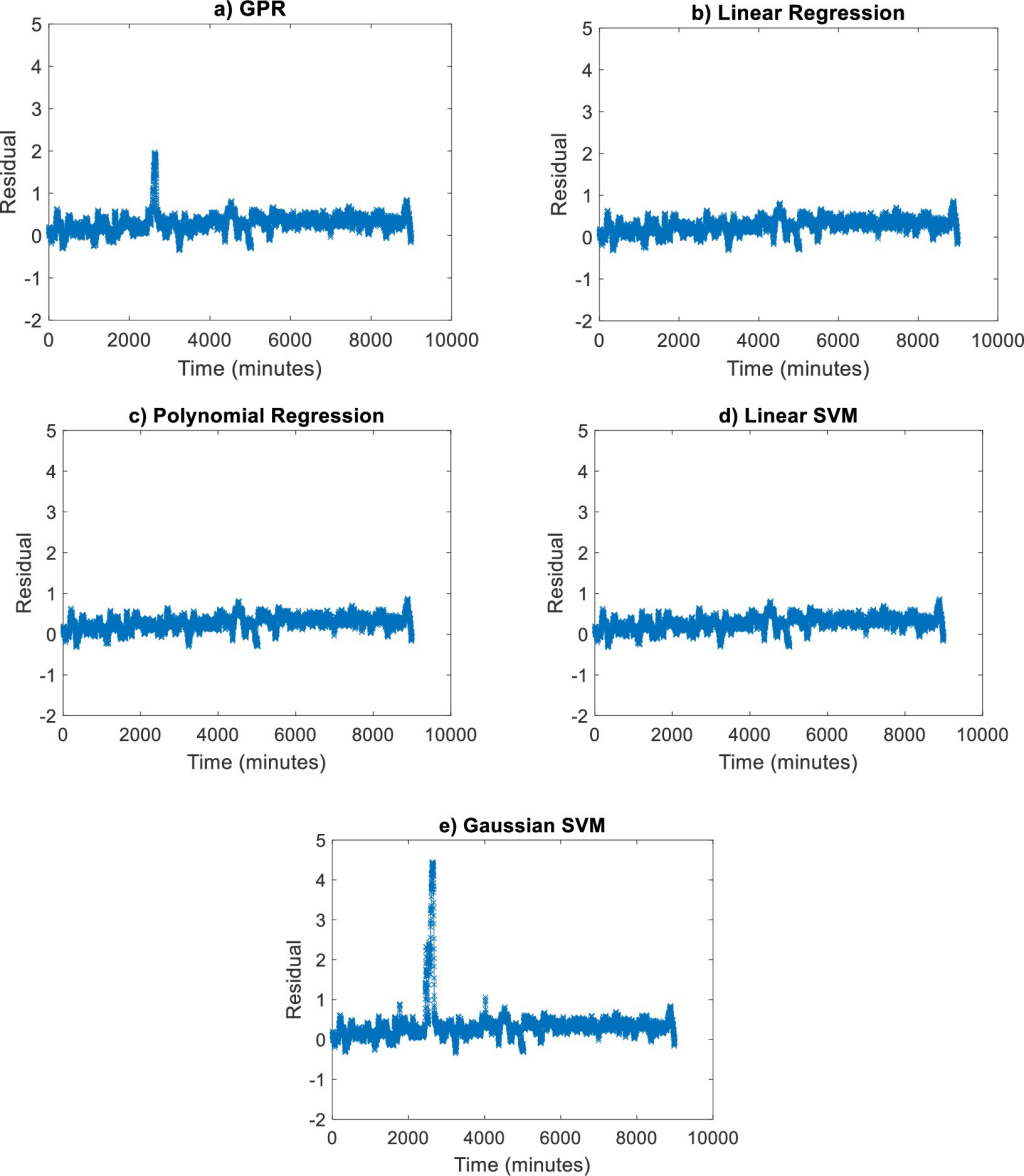
(a)-(e). Residual plot of all estimation techniques for *T*_*h*_ estimation.

Based on the residual plot analysis as illustrated in Fig 11, Linear Regression, Polynomial Regression and Linear SVM are the most equally distributed and small errors between the negative and positive axis. Whereas, GPR and Gaussian SVM produce a high error in the afternoon when the high temperature during that time. This residual pattern indicated a bad fit for the regression. Overall, the model can learn the data very well since the motor temperature is highly related to the winding temperature.

Other than graphical analysis, the methods were evaluated numerically. The result of the analysis is tabulated in Table 4. It can be seen that the R-square analysis of all methods shows a good fit with a value of more than 0.99 except for Gaussian SVM. For MAPE and RMSE evaluations, Linear Regression has the lowest error with 0.286 and 0.322 respectively. The second-lowest error is Linear SVM followed by Polynomial Regression and GPR. However, Gaussian SVM exhibits the highest error of MAPE and RMSE values.

**Table 4:** Comparison between different methods for *T*_*h*_ estimation.

Method	R-square	MAPE	RMSE
GPR	0.913	0.306	0.362
Linear regression	0.931	0.286	0.322
Polynomial regression	0.929	0.290	0.325
Linear SVM	0.930	0.288	0.324
Gaussian SVM	0.801	0.348	0.546

Based on the results of this investigation, Linear Regression is chosen as the best approach for estimating *T*_*h*_ values in the thermal model since it has the highest accuracy when compared to other methods. Moreover, a good distribution of fit estimation from the prediction plot.

**Fig 12:**
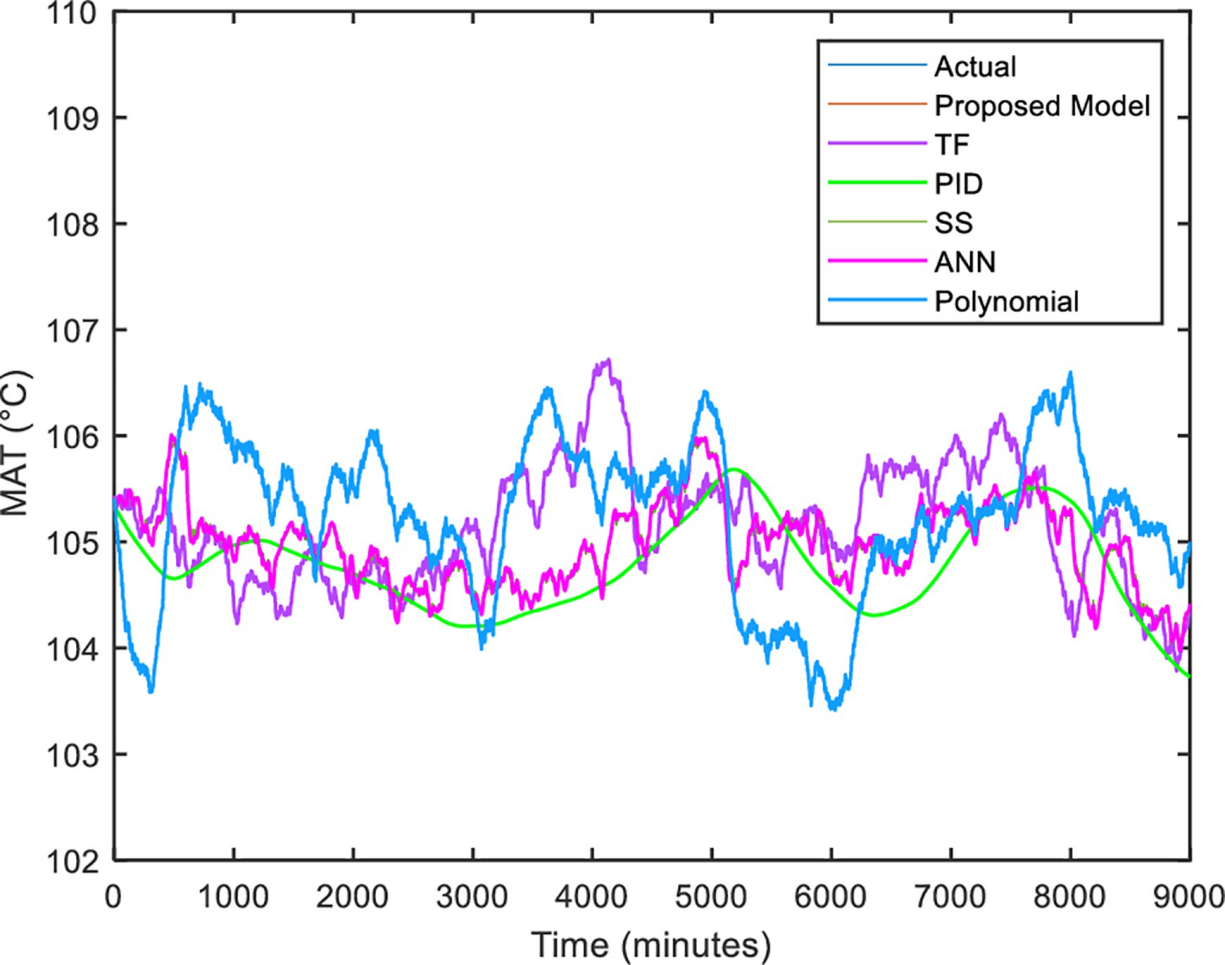
Comparison between actual and simulated hot air temperature, *T*_*h*_.

After the addition of the linear Regression model for *T*_*h*_ calculation into the thermal model, several parametric system identification methods such as transfer function (TF), state-space (SS), proportional integral derivative (PID), polynomial regression and shallow learning of artificial neural network (ANN) [37] are selected to be compared to the proposed thermal model based on mathematical modeling graphically and numerically. The details of the parameters for each method are taken from the existing literature. A plot of the comparison between the thermal models based on mathematical modeling with other methods is illustrated in Fig 12.

The figure above shows the performance of the test data from each method for 10 days of fault-free operation. With closer observation, an identical trend is noticed between the proposed model with actual data. Besides that, it also overlapped with TF and SS models. Both models demonstrate the ability to capture the dynamics of actual data trending. This is because they effectively work in a linear relationship of input and output [38]. However, those models do not help give physical insight into the system. Furthermore, they are not feasible to apply since the disturbance is not considered in the model development and it is difficult to represent the input of disturbance. While ANN cannot capture the motor temperature value due to insufficient data to learn the behavior. The higher-order polynomial regression also did not perform well due to its poor interpolatory properties, resulting in misleading. PID is the most incorrect model because its parameters (the gains of the proportional, integral, and derivative terms) are affected by the process input, causing the output to be unstable and diverge.

In addition to graphical analysis, the developed thermal model is evaluated numerically. The performance of the thermal model developed in Simulink is compared with other methods as illustrated in Table 5. It can be observed that mathematical modeling outperforms linear and nonlinear models. The accuracy of the mathematical model is contributed by the theory of heat transfer and energy balance. Even TF and SS models can capture the thermal dynamics of the HVIM because the RMSE and MAPE are close to the mathematical model, but it is hard to characterize the disturbance of the system.

**Table 5:** Comparison between different thermal models.

Model	MAPE	RMSE
TF	0.636	0.757
SS	0.637	0.757
Polynomial	4.786	4.837
PID	5.637	5.760
ANN	4.703	4.785
Proposed model	0.596	0.614

Furthermore, ANN exhibits higher error since it is unable to predict effectively in a dynamics system with a limited amount of training data. PID and polynomial models also have poor performance in capturing the dynamic of motor temperature with higher errors of MAPE and RMSE. Therefore, the mathematical model is selected to provide hot air temperature data because it is more valuable in studying the dynamics of motor temperature effects and more flexible in modeling the disturbance characteristics.

### C. Cooler model

**Fig 13:**
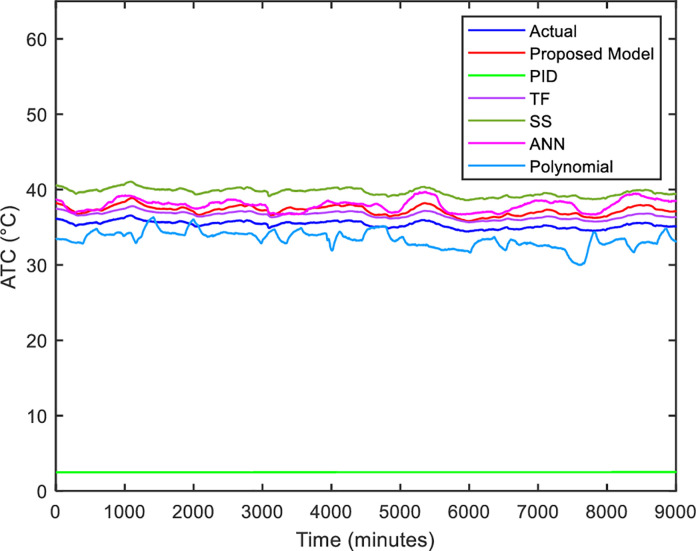
Comparison between actual and simulated cold air temperature, *T*_*c*_.

The motor cooler model acquired actual data of hot air temperature and constant values of the water cooler temperature and flowrate as input. After simulation, the output trend of cold air temperature, *T*_*c*_ is observed. The graphical result shows the simulated responses from all the methods as depicted in Fig 13.

With a thorough observation of the results, the mathematical model developed in Simulink is almost identical to actual data. When the actual *T*_*h*_ data is applied, the output of the *T*_*c*_ parameter follows the trend of actual data. This is due to the physical characteristic’s ability to adapt to the dynamic of the system. Besides that, other methods like ANN, TF, SS and polynomial can capture the trend of *T*_*c*_. It means that these methods can provide a good approximation of the input-output relationship. Although polynomial regression can accommodate a wide range of curvature, the presence of outliers can have a significant effect on nonlinear analysis. Even state space and transfer function methods use simple algebraic expressions, but they are not practical for modeling the motor cooler since they cannot provide information about the internal state of the system.

The simulation output is further analyzed in terms of MAPE and RMSE. The results are tabulated in Table 6. The simulated data from the Simulink model is compared to several system identification models. According to the findings, there is a significant difference between mathematical models with other models. PID was found to be the highest error when it could not capture the actual trend. This could be due to the inability to incorporate the oscillatory response. The different trend that has been observed in the polynomial curve might be due to non-constant error variance. The proposed method using mathematical modeling exhibits the highest accuracy performance when MAPE and RMSE values are the lowest. It justifies the model’s credibility when the model can represent the system very well.

**Table 6:** Comparison between different cooler models.

Model	MAPE	RMSE
TF	1.007	3.745
SS	1.191	4.091
Polynomial	1.293	4.159
PID	958.100	34.134
ANN	0.680	3.216
Proposed model	0.506	2.653

Besides that, this method is preferred because it helps in the explanation of the physical phenomena of the motor cooler as well as the understanding of the real problem situations. Error analysis proves that the simulated signals are accurate enough to represent the real signals.

### D. Combined HVIM model

**Fig 14:**
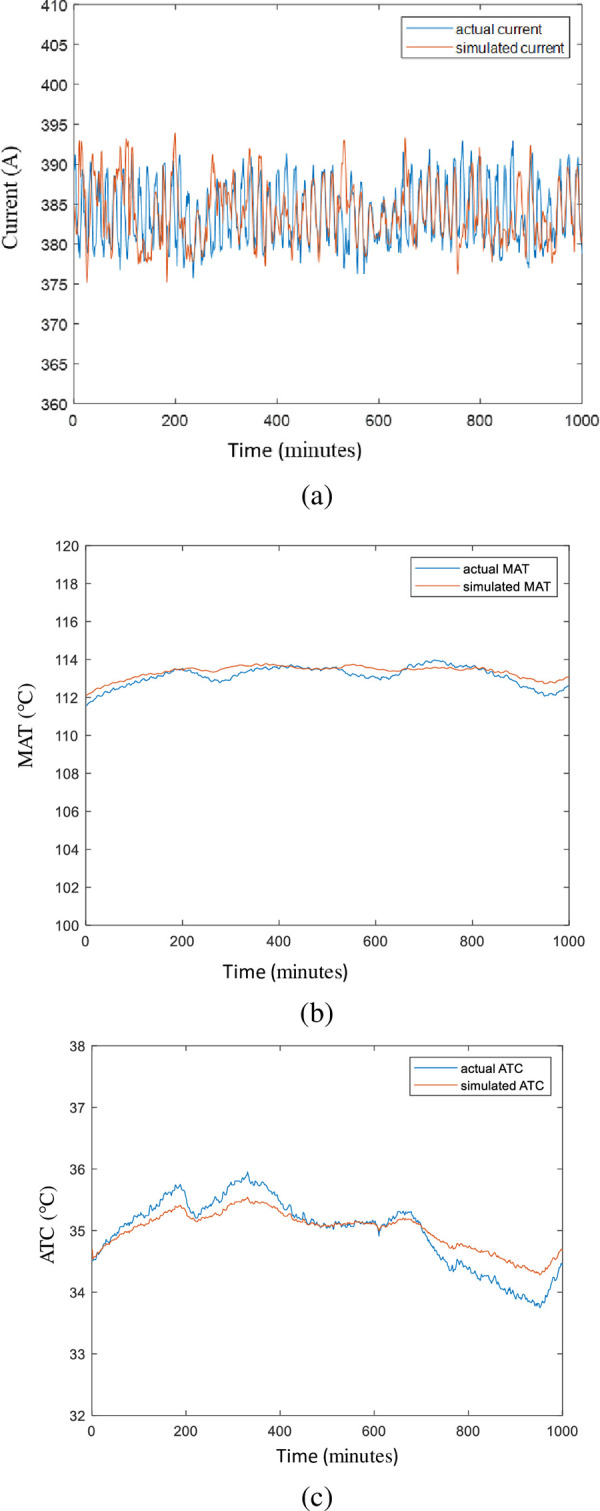
Combined HVIM model results for (a) electrical, (b) thermal and (c) cooler.

After modeling the electrical, thermal and cooler models, these models are combined. The simulated output from the combined HVIM model is compared graphically with the actual signal using healthy data input as shown in Fig 14. With thorough observation, the simulated signals (red plot) successfully captured all the trends of the actual signals (blue plot). However, the thermal and cooler models are slightly deviate compared from the individual model. The difference is caused by the accumulated error from the simulated current. The simulation outputs are further evaluated in terms of RMSE and MAPE. The results of the simulated and actual signals of current, *T*_*h*_ and *T*_*c*_ are tabulated in Table 7.

**Table 7:** Results analysis from the combined model.

Parameter	RMSE	MAPE
Current	4.3405	0.8050
Hot Air Temperature, *T*_*h*_	0.3401	0.2516
Cold Air Temperature, *T*_*c*_	0.5214	0.9964

The slightly higher RMSE and MAPE of the current signals compared to hot air and cold air temperatures are due to the high deviation of the current signals. Furthermore, *T*_*h*_ produced lower error than *T*_*c*_, and the accuracy of *T*_*c*_ performance has slightly deteriorated from the actual signals. These situations occurred due to the cumulative errors from other simulated variables before *T*_*c*_. But, MAPE still produced less than 1% and even had low RMSE. It can be verified that there is low variance among the simulated data and most signals deviate uniformly from the actual signals. This condition proves the actual trend has been captured successfully. Therefore, simulated *T*_*c*_ from the combined model based on mathematical modeling can be utilized for fault diagnosis or prognosis purposes. In general terms, the results obtained in this may serve as useful information to engineers and the industry, especially on the maintenance planning of the induction motor cooler.

## VI. Conclusion

Recently, high voltage induction motor cooling was given attention to prevent motor overheating and catastrophic failure. In the past, an induction motor with internal cooling was designed. However, these two streams have historically developed independently. Since HVIM required an external cooling system, combining analysis of electric circuit design with the thermal analysis of internal and external cooling is a current and emerging concept. Incorporating these aspects at the preliminary design stage will improve the accuracy of model performance for thermal analysis and prediction. In this paper, the mathematical model of the HVIM cooling system is proposed by integrating the cooler and thermal models with the electrical model. There are three major contributions in this paper to represent the HVIM cooling system. First, the individual HVIM model was developed for the electrical, thermal and cooler parts. Secondly, the improvement is made by adding a linear regression method and a modified correction factor to thermal and cooler models respectively. Finally, the novel simulation model of the HVIM cooling system was designed by integrating all the models to represent the real setup of the HVIM cooling system and validated using available actual data from the plant. To verify the proposed method, the simulation output of the current, motor temperature and cold air temperature signals from the individual model were compared with the actual signals from the power plant. In terms of error analysis, the MAPE of current is 0.872, *T*_*h*_ is 0.5964 and *T*_*c*_ is 0.5095. The combined HVIM model also is proven accurate because the errors do not exceed the industry’s acceptable limit of 1%. The results clearly demonstrated the accuracy of the proposed model, with very low MAPE and RMSE. We believe that this combined HVIM mathematical model can be applied in condition monitoring, fault diagnostics and prognostics studies. To further improve the utilization of the model, water cooler heat transfer, temperature of winding, and bearing can be added and the failure mechanism of the cooling system can be developed in future research. So, the work can appropriately be employed to predict the temperature distribution and thermal failure of the induction motor cooler.
